# De-anthropomorphizing brain mapping: How a “component” perspective unbounded by behavioral categories may reconcile localization vs. circuit-based models of brain function

**DOI:** 10.3389/fnsys.2022.946715

**Published:** 2022-07-27

**Authors:** Anne J. Blood

**Affiliations:** Mood and Motor Control Laboratory, Department of Psychiatry and Neurology, Psychiatric Neuroimaging Division, Athinoula A, Martinos Center for Biomedical Imaging, Harvard Medical School, Massachusetts General Hospital, Boston, MA, United States

**Keywords:** behavior, brain mapping, modular, circuit, impedance, component, organizational principles

## Introduction: Historical and current approaches to mapping brain function

Models of brain disorders often propose that symptom pathophysiology reflects disorganization of neural programs, either locally or at the circuit level (e.g., Lambert et al., 2018; Uehara et al., 2019). Indeed, *pathogenesis* of brain disorders (e.g., stroke, genetic mutations) frequently does not proceed in an organized fashion. However, symptoms and/or behaviors in these disorders are often stereotyped, which is how we have been able to identify and classify them clinically. So why would disorganization in *pathophysiology* of specific symptoms (if defined as the “encoding” of such symptoms) emerge as something stereotyped which, conceptually, is the opposite of disorganized (Blood, 2008)? The idea that structural or functional brain disorganization directly translates to the behavioral dysfunction we observe seems consistent with a belief that the underlying functional units of the brain correspond with healthy behaviors as we see and define them. In other words, we have anthropomorphized the way we believe the brain is organized. What if we instead use the stereotyped nature of symptoms themselves as a clue to alternate ways of viewing brain organization? What if the qualitative features of disorder symptoms that allow us to classify them reflect abnormal amplification or attenuation of some pre-programmed function in the brain—a normal, healthy unit of function, not a disorganized, “unwanted,” or “scrambled” one, but also one that is not itself a discrete behavior?

## Clues to alternative explanations and mechanisms

In my lab's research we have taken the perspective that stereotyped behavioral abnormalities may provide clues to brain systems or “components” that integrate into full-scale behavior (Blood, 2008, 2013). We will use dystonia as an example here. Dystonia is stereotyped; it has a distinct clinical appearance and frequently involves co-contraction of antagonistic muscles. This co-contraction is thought to be a qualitative error in output—a failure to disinhibit competing motor programs—but then the resulting output should be random and unpredictable, not stereotyped. Might co-contraction at a low amplitude serve some functional purpose in normal, healthy movement/motor control? One possibility, from an engineering/mechanical perspective: Co-contraction provides a mechanical “impedance” signal that could implement a broad array of body stabilization and motor speed/precision functions that are desired during movement rather than inhibited. In previous papers we proposed that the brain implements movement by calling upon a combination of stored basic motor programs (direct pathway) and neural impedance programs (indirect pathway) that are used in a variety of postural functions but which are also used in a much broader manner than what we traditionally define as “posture” (Blood, 2008, 2013). Dystonia, according to this hypothesis, reflects vast overamplification of an impedance signal. We propose at least four different subtypes (or “modules”) of impedance function in the brain subserving global and local body stability, speed/precision control, and baseline muscle tone, each of which might be distinctly encoded in a different brain motor cortical or cerebellar region. The basal ganglia are hypothesized to coordinate which of these components are needed for a particular behavior. Mapping this concept back onto the clinical presentation of dystonia, including qualitative variation in presentation, we proposed that the brain may use the principle of impedance in numerous ways across brain regions to implement numerous behavioral functions, but that (to quote from Blood, 2013) “this system is actually best characterized by the functions it performs, rather than by subjective descriptions of behavior in which we see its manifestations (e.g., posture).” The “component” perspective has come up in other areas of neuroscience, including learning and memory research (e.g., Witherspoon and Moskovitch, 1989; Moscovitch, 1992; Cabeza and Moscovitch, 2013). What I propose here is complementary to other models but differs in asserting that component categories or mechanisms may be unbounded by behavioral functions or categories as we experience them. It also allows encoding to be simultaneously modular and domain general. For example, in the case of the proposed impedance component there is evidence from a symptom/conceptual perspective that the same impedance mechanism is used to modulate more complex behaviors and not only motor control.

The component perspective is consistent with a more general principle recognized in other areas of science such as physics: that complexity can emerge from simple building blocks and that such simplicity leads to greater economy and flexibility when building a complex system (Wilczek, 2015). Might behavior be an emergent property of components that come together seamlessly, through millennia of refinement, but which aren't in and of themselves the “essence” of the behavior, much as steel and rubber do not become a car unless they are put together in a certain way, and each can serve many other functions? In healthy control populations it would be very difficult, if not impossible, to resolve the individual components because they are normally so seamlessly integrated they do not appear to be distinct functions. This bears conceptual similarity to the hidden organizational features of the brain proposed by Doyle and Csete (2011). In the case here, the proposed features are control functions that should, in fact, be measurable in behavioral output if the proper variables are known (i.e., they appear hidden only because they are not resolvable as discrete behaviors as *we* categorize them). In brain disorders, stereotyped behavior gives us clues to the building blocks I propose here. Behaviors that seem “odd” may simply be individual components that appear unusual on their own, when not in correct proportion to the other components of a behavior. Disorders have obviously long been studied for clues to underlying systems; what I suggest here is a subtle, but significant shift in how we might view and interpret such clues. This may be an especially important vantage point to consider when studying mechanisms underlying circuit- and/or dimensional-based understanding of neuroscience—that is, how do multiple brain regions construct a single function or behavior and why are multiple regions required?

It is important to note that the idea of using engineering principles to understand the brain is not new, nor am I suggesting all components of behavior use engineering principles. What is novel here is that rather than *a priori* selecting a specific engineering principle, approach, or algorithm to search for clues to organization, we have observed evidence of engineering principles in unconstrained data. Other such components likely have already been identified and I also do not intend to suggest *a priori* engineering approaches are not valuable for discovery of new organizational principles; this paper is simply a call to consider this additional way of detecting and thinking about brain organization when designing and interpreting future studies.

In summary, this suggests when studying how the brain constructs behavior we should consider that each brain region of a functional circuit may contribute its own component to the behavior, but no component will resemble any behavioral feature we could intuit just by observing normal behavior. If this is the case, required components for a given behavior would need to work together harmoniously for the desired behavior to occur; this idea is consistent with altered functional connectivity (FC) frequently observed in brain disorders (e.g., Fox et al., 2012a,b; Servaas et al., 2015; Schultz et al., 2017; Maiti et al., 2020; Hua et al., 2021). Abnormal behaviors may not result from altered circuit synchrony itself, changes in synchrony may instead signal excessive or reduced output of a single behavior component. To the extent that components do not come together in the correct proportions or timing one could argue the behavior was disorganized. But it does not mean the component programs themselves are disorganized. One could argue this is merely a semantic issue but the implications of the proposed mechanism are that we should not necessarily be thinking that coding of a particular program is disorganized when we see an unusual behavior; that is, no “new behavior” or “new component” was created.

## Discussion

### Reconciling modularity vs. distributed circuit encoding of function

The evidence above suggests individual behaviors may emerge by combining simpler units of function (“components”) which themselves are not in any way unique to that behavior. Conversely, each component may contribute to multiple behaviors. Importantly, this concept is consistent with a hybrid of localization/modular and circuit-based approaches to neuroscience (see Mather et al., 2013 for previous debate on this topic) and argues that each approach is valid and that they complement, rather than compete with each other. Specifically, it suggests there may be localization of particular functional “components” but multiple components must be assembled across multiple brain regions (or at least multiple neurons within a region) in a temporally synchronous manner to construct a whole behavior. Thus, behavior emerges from the orchestrated activity of a circuit, but each region within the circuit has its own unique and independent function. This approach is appealing from an encoding burden perspective since it requires less overall information be encoded by the brain—complexity emerges from how components are combined rather than from complexity of individual programs. In our specific example, above, motor complexity emerges from basic functional units of movement combined with mechanical impedance units that not only keep behavior “under control” but also sculpt the qualitative nature of that behavior (precision, speed, gracefulness, etc.). Note that the proposed impedance components can modulate behavioral qualities in an almost infinite number of ways merely by changing the amplitude of the system output or by varying which impedance component is used.

The ideas discussed here arose from studying behavior specifically (or “system output” to avoid anthropomorphizing) so it is not yet clear whether it applies to perception and cognition (“input” and “processing”); it is quite possible that different brain systems evolved with different organizing principles. These ideas also remain consistent with the possibility that some brain functions are so evolutionarily conserved or critical for survival [e.g., reflexes, perception of items such as faces (e.g., Kanwisher et al., 1997)] that some components are closer to behavior or perceptions as we see and classify them. In other words, this is not intended to be a unifying theory of how the whole brain works, just one additional—but critical—piece of the puzzle.

### Implications for systems neuroscience, including brain imaging studies

In addition to reconciling modular and circuit models of brain function, the proposal that the brain uses non-behavior-equated components to orchestrate behavior may yield several other advances in systems neuroscience research. For example, clues to the brain's operative variables will lead to more targeted task paradigm design aimed at parsing units of function. More targeted paradigm design will lead to better replication of findings across functional imaging studies; seemingly minor shifts in paradigm across studies in the past may have elicited more substantial differences in brain activation than expected if the shift bridged multiple functional systems (i.e., the shift may not have been as minor as we believed). Thus, with more targeted design we may identify insightful biological reasons for past cross-study discrepancies, rather than methods reliability problems. As alluded to above, the “component” concept of behavior construction also suggests a specific, mechanistic interpretation for functional connectivity MRI (FC-MRI) studies and the relevance of functional synchrony to “assembling” a behavior correctly.

Finally, it is important to emphasize that operative variables need not be known to design useful or important functional imaging studies; discovery, non-hypothesis-based studies are essential in narrowing the field to detect the operative variables the brain uses and every new piece of information in a well-designed hypothesis-driven study provides clues that might identify organizational principles in the future (Mitra, 2014; Calhoun and Bandettini, 2020). Indeed, some of the concepts we eventually gleaned from behavior were hinted to us by unexpected data-driven findings with task fMRI (Blood et al., 2004). The proposed view of behavior “assembly” is merely meant to be another step in our evolving understanding of the brain.

## Author contributions

AB is the sole contributor to this publication, including conception of the idea, and writing of the manuscript. She has approved the final version of the article.

## Funding

Although this publication was written without specific financial support, previous work under Grants R21NS046348 and R01NS052368 from NINDS and two Grants from the Dystonia Medical Research Foundation (DMRF) supported research that led to the generation of the concepts presented here.

## Conflict of interest

The author declares that the research was conducted in the absence of any commercial or financial relationships that could be construed as a potential conflict of interest.

## Publisher's note

All claims expressed in this article are solely those of the authors and do not necessarily represent those of their affiliated organizations, or those of the publisher, the editors and the reviewers. Any product that may be evaluated in this article, or claim that may be made by its manufacturer, is not guaranteed or endorsed by the publisher.
